# Supplementary material for: Novel Immunoregulatory Functions of IL-18, an Accomplice of TGF-β1
Source: Cancers (Basel). 2019 Jan 11;11(1):75. doi: 10.3390/cancers11010075 (PMC6356463; doi:10.3390/cancers11010075)
Supplement: Supplementary file 1 [file cancers-11-00075-s001.pdf]

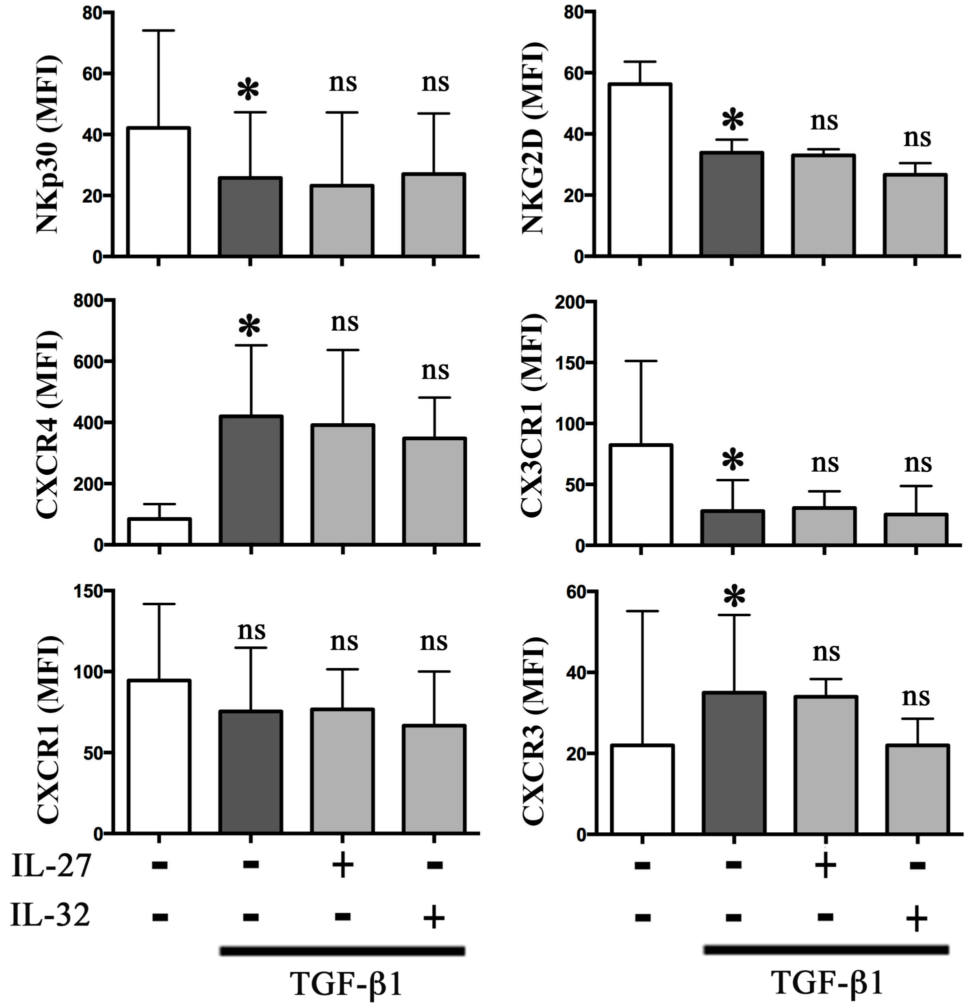
NKp30 (MFI)
NKG2D (MFI)
CXCR4 (MFI)
CX3CR1 (MFI)
CXCR1 (MFI)
CXCR3 (MFI)
*
ns
IL-27
IL-32
TGF-β1

Figure S1

IL-27 or IL-32 does not influence the TGF-β1-mediated immunomodulatory effects.

Cytofluorimetric analysis of the expression of activating and chemokine receptors in PB NK cells cultured for 48 hours in the absence or in the presence of TGF-β1 used alone or in combination with the indicated cytokines. Average of 6 independent experiments (6 donors). Mean of MFI and 95% confidence intervals are shown. ns: not significant. Statistical significance is referred to CTR.

Mean of MFI and 95% confidence intervals are shown. ns:not significant, *p<0.05.

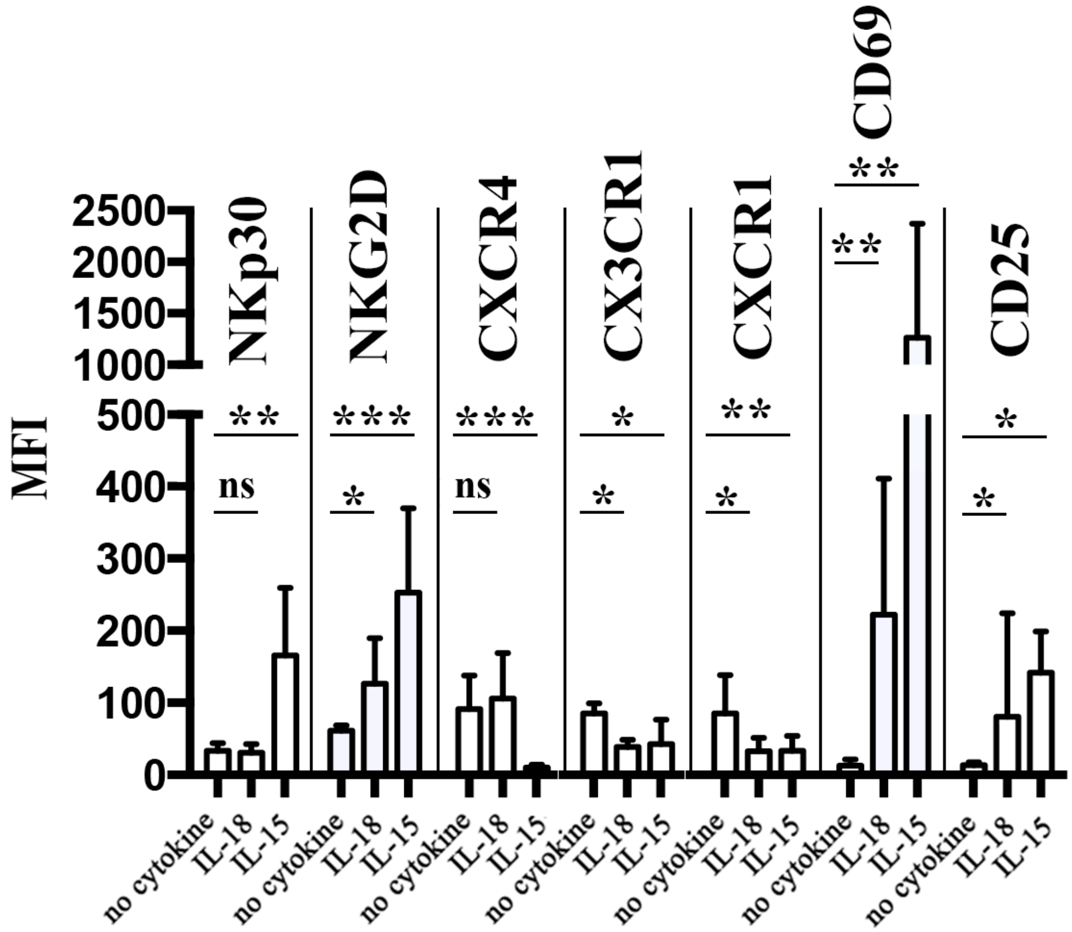

MFI
2500
2000
1500
1000
500
400
300
200
100
0
NKp30
NKG2D
CXCR4
CX3CR1
CXCR1
CD69
CD25
ns
*
**
***
no cytokine
IL-18
IL-15

Figure S2

Effect of IL-18 or IL-15 on activating and chemokine receptors expression

PB NK cells, cultured for 48 hours either in the absence or in the presence of IL18 or IL-15, were analyzed by flow cytometry for the expression of the indicated molecules. Average of 6 independent experiments (6 donors). Mean of MFI and 95% confidence intervals are shown. ns:not significant, *p<0.05, **p<0.01; ***p<0.001.

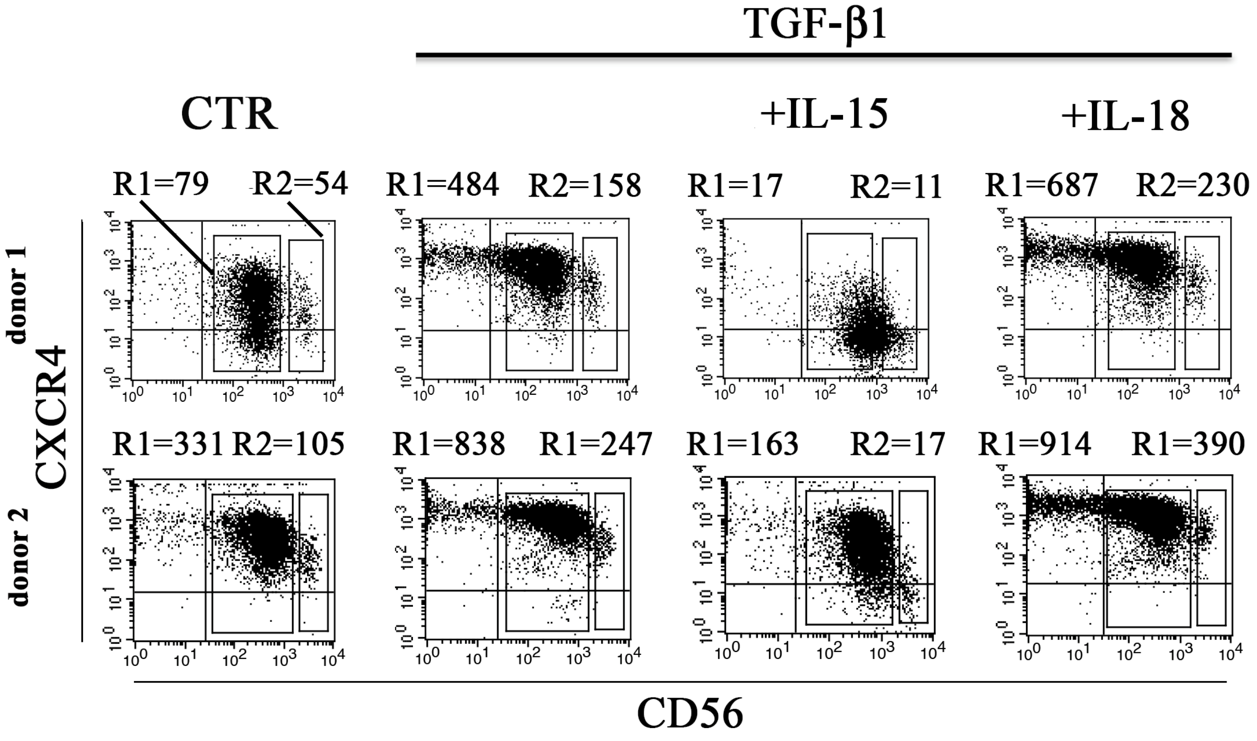

Figure S3

IL-18 increases the TGF-β1-mediated CXCR4 up-regulation in both CD56^{dim} and CD56^{bright} NK cells.

CD56^{dim} (R1) and CD56^{bright} (R2) PB NK cells, untreated (CTR) or treated with the indicated cytokines, were analyzed for the expression of CXCR4 by double color flow cytometry. Results from two representative healthy donors are shown. R1 and R2 values indicate MFI.

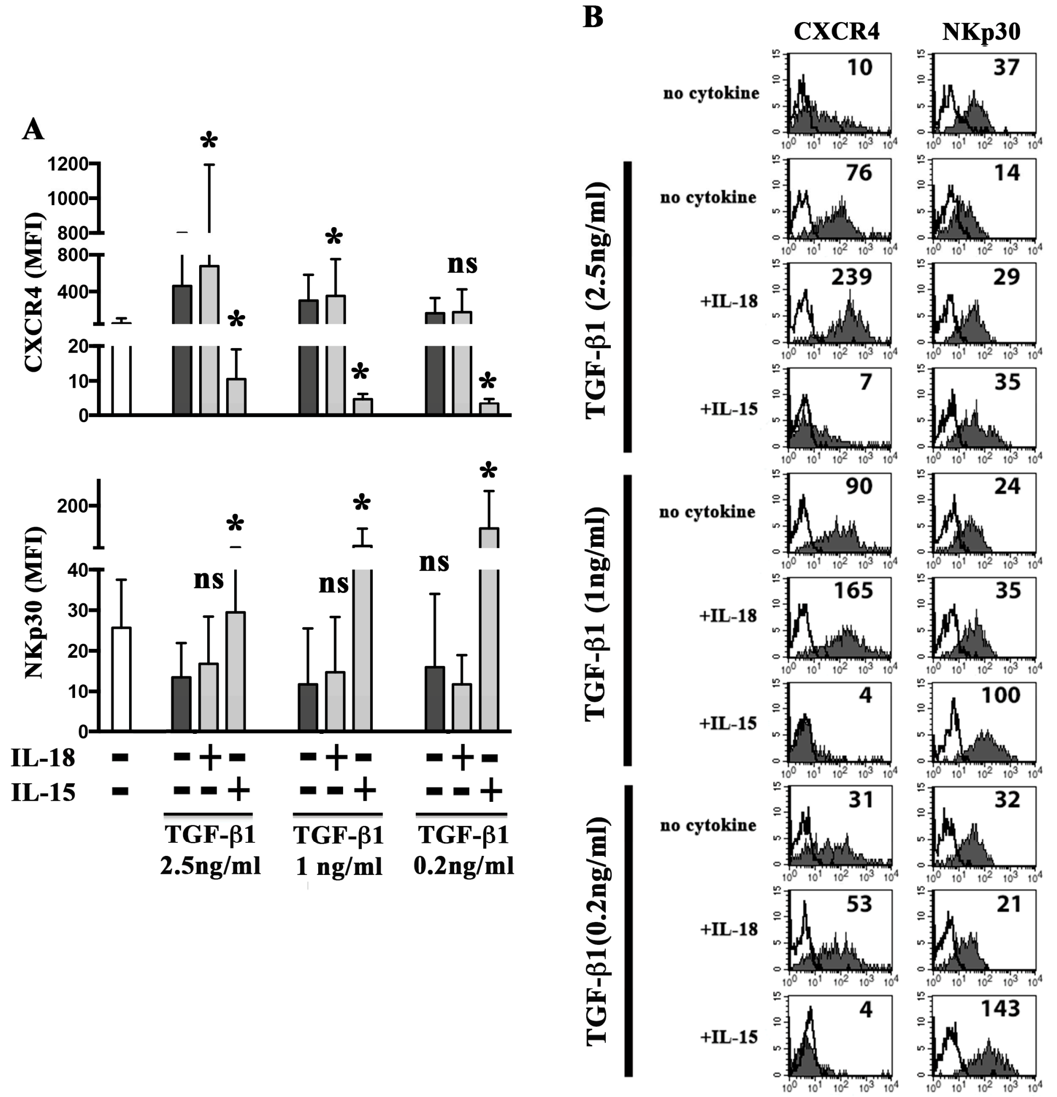

Figure S4

Analysis of the effects of IL-18 in combination with sub-optimal doses of TGF-β1

PB NK cells, cultured for 48 hours in the absence or in the presence of decreasing doses of TGF-β1 alone or in combination with IL-18 or IL-15, were analyzed by flow cytometry for CXCR4 and NKp30 (panel A). Average of 5 independent experiments (5 donors). Mean of MFI and 95% confidence intervals are shown. *$p<0.05$; ns means p not significant. Statistical significance is referred to results obtained with TGF-β1 alone. Representative experiment is shown in panel B. White profiles refer to cells incubated with isotype-matched mAbs. Values inside each histogram indicate the MFI.

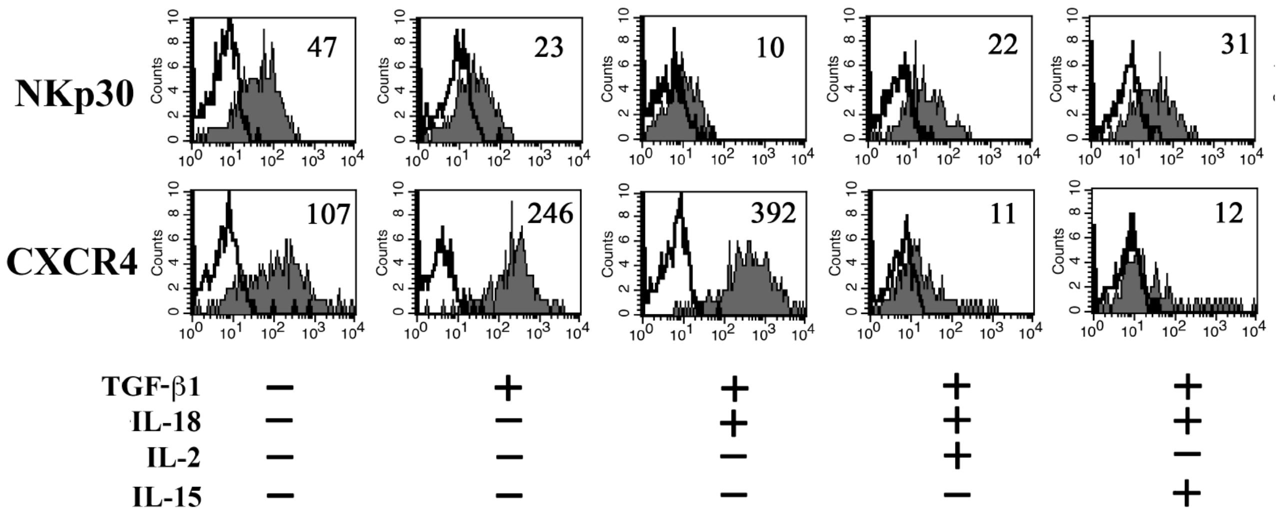

Figure S5

IL-2 or IL-15 contrasts the immune-modulatory effects mediated by TGF-β1 plus IL-18.

Representative experiment of the cytofluorimetric analysis shown in Fig.2, panel B. White profiles refer to cells incubated with isotype-matched mAbs. Values inside each histogram indicate the MFI.

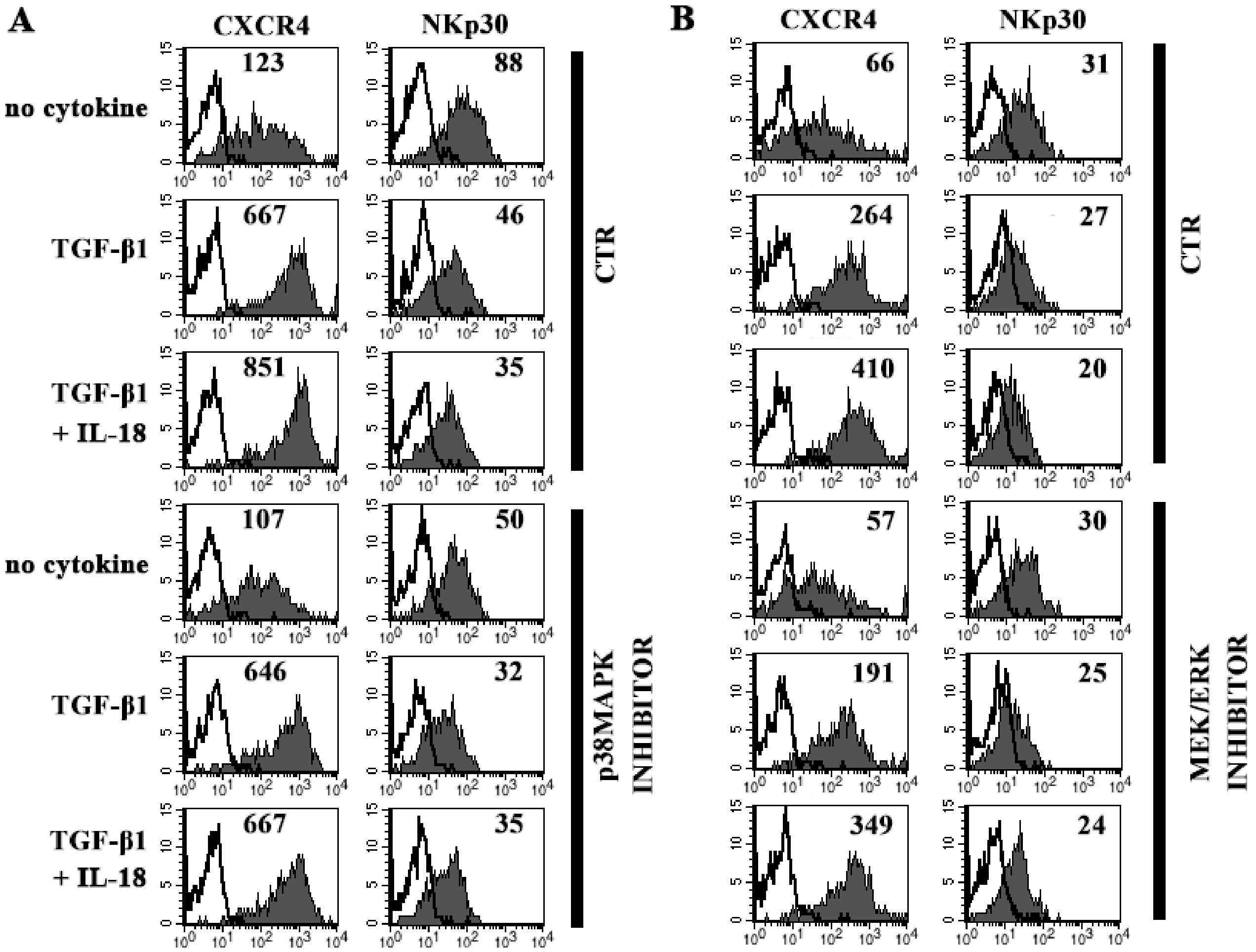

Figure S6

Effect of p38MAPK or MEK/ERK inhibitors on the IL-18 additive effects

Representative experiment of the cytofluorimetric analysis shown in Figure 4. White profiles refer to cells incubated with isotype-matched mAbs. Values inside each histogram indicate the MFI

Proteomic analysis

The samples were processed by in-Stage Tip method using enclosed StageTip, containing 1 SDB-RPS disks. Briefly the pellets were solubilized in 25 μL of 6 M GdmCl. 10 mM TCEP, 40 mM CAA, 100 mM Tris pH 8.5. In a single step the cells were lysated, reduced and alkylated by three cycles of heat and thaw and by sonication for 30 min and then loaded into StageTip. The lysates were diluted with 10% (v/v) ACN, 25 mM Tris pH 8.5 and digested with 0.5 μg of lysine C and 0.5 μg trypsin at 37 °C overnight. The samples were acidified with 100 μl of 1% (v/v) TFA and washed three times with 0.2 % (v/v) TFA. Elutions were performed with 60 μl of 5% (v/v) ammonium hydroxide, 80% (v/v) ACN.

Peptide separation was performed with Dionex Ultimate 3000 RSLC nanoSystem. Samples were first loaded from the sample loop onto a trapping column (2 cm × 100 μm ID, Acclaim PepMap C18, 2 μm particles, 100 Å pore size; Thermo Scientific Cat. No. 164564) using the loading solvent (98% H2O and 2% CH3CN, 0.1% formic acid) at a flow rate of 5 μl/min for 5 min. The trapping column was then switched in-line with the separation column, and peptides were eluted with increasing organic solvent at a flow rate of 300 nl/min. The peptide separation was carried out using EASY-Spray column (25 cm x 75 μm ID, PepMap C18, 2 μm particles, 100 Å pore size; Themo Scientific Cat. No. ES803), mounted on the EASY Spray Ion Source, thermostated at 60 °C. Peptides were separated and eluted with a multi-steps gradient of 5–30% solution B (80% CH3CN and 20% H2O, 0.1% formic acid) in 170 min, 30-45% solution B in 35 min.

The mass spectrometer LTQ-Orbitrap Velos Pro was operated in positive ionization mode. Single MS survey scans were performed in the Orbitrap, recording a mass range between 375-1500 m/z with a resolution of 100000. The automatic gain control was set at 1.000.000 with a maximal ion injection time of 250 ms.. The experiments were done in data-dependent acquisition mode with alternating MS and MS/MS experiments and using the preview mode for FT master scan option. The minimum MS signal for triggering MS/MS has been set to 10000 ions, with the most prominent ion signal selected for MS/MS using an isolation window of 2 Da. The m/z values of signals already selected for MS/MS were put on an exclusion list for 25 s using an exclusion window size of ±10 p.p.m. In all cases, one micro-scan was recorded. CID was done with a target value of 3,000 ions in the linear ion trap, a maximal ion injection time of 50 ms, normalized collision energy of 35%, a Q-value of 0.25 and an activation time of 10 ms. A maximum of 10 MS/MS experiments were triggered per MS scan.

Raw mass spectrometric data were analyzed with the MaxQuant software (version 1.6.1.0). A false discovery rate (FDR) of 0.01 for proteins and peptides and a minimum peptide length of 6 amino acids were required. A time−dependent mass recalibration algorithm was used for recalibration to improve the mass accuracy of precursor ions. MS/MS spectra were searched by the Andromeda search engine, which is incorporated into the MaxQuant software suite against Uniprot Human database (release 2017_08) combined with 248 common contaminants and concatenated with the reversed versions of all sequences. The trypsin was chosen as enzyme specificity. Cysteine carbamidomethylation was selected as a fixed modification, while protein N-terminal acetylation, methionine oxidation and deamidation (N, Q), were selected as variable modifications. Maximally two missed cleavages were allowed. Initial mass deviation for the precursor ion was up to 7 ppm, and maximum allowed mass deviation for fragment ions was 0.5 Da. Quantification in MaxQuant was performed using the built-in label-free quantification algorithm MaxLFQ enabling the 'Match between runs' option (time window 1 minutes).
